# Using wastewater surveillance to improve infectious disease control in correctional facilities and congregate living settings: A modeling perspective

**DOI:** 10.1016/j.epidem.2026.100898

**Published:** 2026-02-14

**Authors:** Daniel de-la-Rosa-Martinez, Kara L. Nelson, Joyce Lee, Rose S. Kantor, Ashley Hazel, Seth Blumberg

**Affiliations:** a Francis I Proctor Foundation, University of California, San Francisco, CA, USA; b Department of Civil and Environmental Engineering, University of California, Berkeley, CA, USA; c Physical and Life Sciences Directorate, Lawrence Livermore National Laboratory, Livermore, CA, USA; d Department of Medicine, University of California, San Francisco, CA, USA

**Keywords:** Wastewater surveillance, Infectious disease modeling, Congregate settings, Correctional facilities, Outbreak detection

## Abstract

Wastewater surveillance is a valuable tool for monitoring infectious disease dynamics. However, its integration into outbreak control strategies in congregate settings requires further exploration. As observed during the SARS-CoV-2 pandemic, these high-risk environments can facilitate large outbreaks, further exacerbated by residents’ heightened vulnerability. Congregate settings exhibit distinct epidemiological dynamics that influence wastewater surveillance. For instance, their semi-closed populations and reduced mobility can lower environmental noise in wastewater signals, but small population sizes also increase stochastic fluctuations, complicating the interpretation of disease trends. In this context, mathematical modeling helps translate wastewater signals into actionable insights for outbreak response. This work synthesizes key benefits and challenges in applying wastewater surveillance in congregate settings and identifies modeling approaches that have potential to improve outbreak detection, enhance monitoring of transmission dynamics, and optimize infection control strategies. This provides a conceptual framework for expanding wastewater surveillance to strengthen infectious disease control in these high-risk populations.

## 1. Introduction

Wastewater surveillance has been successfully used to monitor a wide range of public health concerns, including vaccine-preventable diseases, recreational drug use, antibiotic resistance, emerging pathogens, and other infectious agents in community settings (Kilaru et al., 2023; Zarei et al., 2020). This approach enables the detection of specific substances or pathogens contributed by a population in a specific geographic area that drains into the sampling site (Kilaru et al., 2023). A significant advantage is that wastewater data are independent of the proportion of clinically tested individuals and include information about asymptomatic carriers (Kilaru et al., 2023).

Congregate living settings, including nursing homes, long-term care facilities, and correctional centers, are characterized by localized groups of residents with daily close contact and limited mobility. In these environments, high population density, shared spaces, and frequent interpersonal interactions amplify the risk of infectious disease transmission, while the high burden of underlying health conditions further increases residents’ vulnerability to severe outcomes (Kendig et al., 2024; Manis et al., 2023; Nicolle, 2014). These health conditions and the structural characteristics of congregate environments can facilitate large outbreaks and increase morbidity and mortality from communicable diseases (Lau-Ng et al., 2020).

Outbreaks in congregate settings can extend beyond these environments, impacting the general population, as infected staff and visitors can seed disease spread in their local communities (Barnert et al., 2020). This risk is particularly pronounced in long-term care facilities, where individuals with severe disease, comorbidities, or compromised immune systems may exhibit prolonged pathogen shedding (Arfijanto et al., 2023). Thus, implementing effective surveillance and mitigation strategies for infectious disease outbreaks is critically important in these populations. This perspective reviews the current state of wastewater-based surveillance in congregate living settings, identifies key challenges, and discusses mathematical modeling approaches for wastewater data analysis to enhance surveillance, outbreak detection, and control response.

### 1.1. Applications of wastewater surveillance for disease control in congregate settings

Wastewater surveillance in congregate settings represents a promising tool for assessing disease burden and guiding the implementation of targeted control measures (Maryam et al., 2023). Although interest in wastewater surveillance in congregate settings has increased in recent years, the number of published studies remains limited, and more applied research is needed to demonstrate its value for decision-making during outbreaks.

Recent works in wastewater-based epidemiology for community settings have identified several challenges, including variability in viral shedding rates, signal underestimation due to dilution and degradation within the sewer system, and integration of wastewater data with conventional surveillance results (Chen et al., 2024). Although several of these limitations also apply to small, semi-closed populations, congregate living environments present valuable opportunities for implementation. Overall, these include reduced variability in environmental features, decreased dilution and viral degradation due to shorter travel distances, and decreased mobility patterns that potentially improve wastewater signal stability. Similarly, the localized scale of congregate facilities enables the collection of high-resolution data, which can be used to improve the accuracy of disease burden estimation (Maryam et al., 2023; Sims and Kasprzyk-Hordern, 2020). Additionally, wastewater surveillance has been well-received by residents and institutional staff as a non-invasive surveillance tool, particularly since testing refusal rates can be high due to stigmatization and fear of isolation (González-Montalvo et al., 2023; Liu et al., 2022).

Despite the differences and potential advantages in semi-closed populations, several challenges remain. Regarding training data, in most scenarios, many cases are unobserved due to inaccurate diagnostic testing or surveillance strategies that fail to capture asymptomatic carriers (Beaudry et al., 2020). Second, stochastic variability in individual-level characteristics such as the timing and magnitude of fecal shedding contributes to increased wastewater signal noise in small communities (Rioux et al., 2023; Sims and Kasprzyk-Hordern, 2020). Third, external wastewater contributions from facility staff, visitors, and neighboring communities introduce additional complexity for interpretation (Sims and Kasprzyk-Hordern, 2020). Fourth, wastewater data are not collected continuously, leading to potential gaps in pathogen detection (Wade et al., 2022). Fifth, high population density and frequent close contact can drive rapid transmission, resulting in sudden increases in wastewater concentrations that are not typically observed in larger populations.

In small congregate settings, an essential consideration for wastewater applications is the combined impact of stochasticity and uncertainty, as random fluctuations can disproportionately affect aggregate estimators and complicate parameter estimation (Rioux et al., 2023; Sims and Kasprzyk-Hordern, 2020). For example, fecal shedding is a significant contributor to this variability, as it is a highly heterogeneous process influenced by a complex interplay of factors, including disease severity, host immune response, vaccination history, prior infections, access to treatment, and pathogen variants (Puhach et al., 2023). As a result, individuals do not contribute homogeneously to wastewater signals, which complicates the establishment of reliable thresholds to inform potential outbreaks.

Given these effects, wastewater analytic approaches should incorporate methods that explicitly account for system variability and uncertainty, including the use of overdispersed frameworks such as branching processes with negative binomial offspring distributions to capture transmission heterogeneity (Lloyd-Smith et al., 2005); bootstrapping methods to generate plausible ranges when small populations and limited sampling constrain inference (Mostofian and Zuckerman, 2019); and Bayesian inference to incorporate prior information on relevant biological and epidemiological processes (e.g., shedding patterns and viral decay), stabilizing estimates and producing more realistic uncertainty bounds in these settings (McMahan et al., 2021).

Facility-specific environmental factors complicate the standardization of analytical approaches, as variables such as temperature, facility layout, sewer flow dynamics, precipitation runoff, and wastewater composition, including contaminants or inhibitors, may influence signal stability and detection accuracy (Kumar et al., 2022; Wade et al., 2022). Studies in correctional institutions have shown that fluctuations in pH and suspended solid levels impact SARS-CoV-2 wastewater signals (Jobling et al., 2024). However, evidence regarding their utility as normalization markers remains inconsistent, and no clear consensus supports their use to improve detection accuracy across settings (Boogaerts et al., 2024).

In addition to environmental variability, uncertainty surrounding the contributing population size can also challenge the interpretability of signals. In response, dietary biomarkers, such as pepper mild mottle virus, are commonly used as proxies for population size within a sewer catchment (Boogaerts et al., 2024). Although these biomarkers offer valuable insights, their reliability may be compromised when they depend on dietary components that are not consistently consumed, particularly in settings with standardized meals. Facilities should therefore prioritize biomarkers that best reflect the actual dietary patterns of their populations (Hsu et al., 2022).

Sampling method and frequency are other critical determinants of pathogen detection in wastewater. Although low-frequency sampling (e. g., once per week) has been shown to provide early warning signals successfully, a reduced frequency may limit the ability to detect abrupt transmission events (Róka et al., 2021; Wade et al., 2022). For instance, grab samples, which reflect a single point in time, may miss pathogens when prevalence is low, whereas time-based composite sampling, which collects wastewater over a defined period, can potentially provide a more representative signal (Wade et al., 2022).

These implications are particularly important in congregate settings where wastewater flow is low and discharge events occur at specific times of the day (Wade et al., 2022). Capturing a representative signal may require continuous or more frequent sampling strategies in such contexts. Equally important is the timely processing and reporting of data, as delays can undermine the benefits of early detection and reduce the utility of wastewater monitoring for rapid outbreak response.

**Fig. 1.**
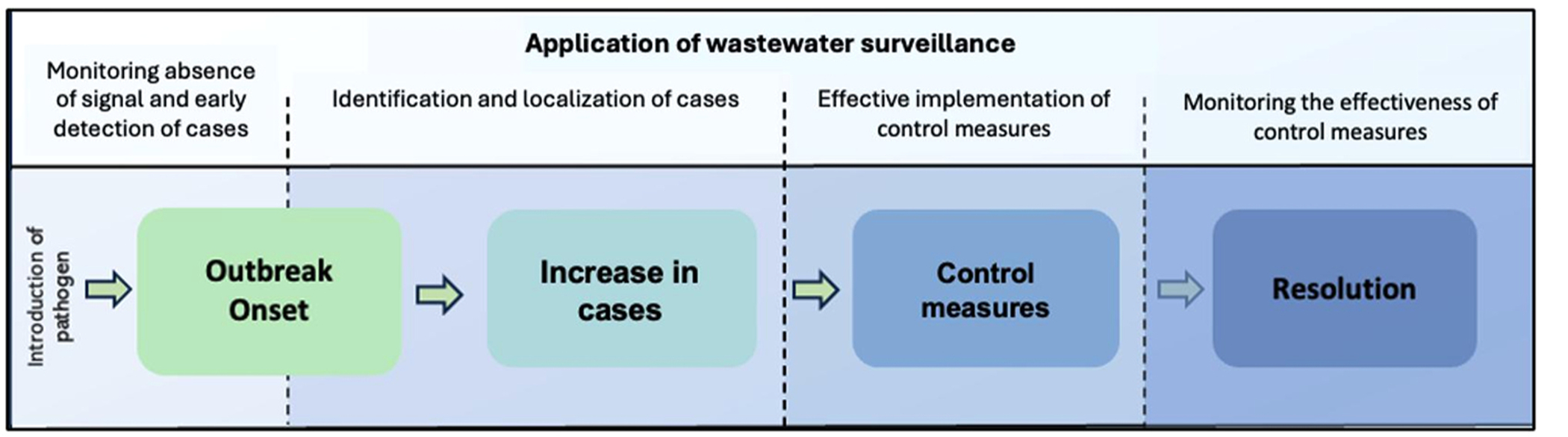
Stages of outbreak dynamics and applications of wastewater surveillance for disease monitoring.

Considering this potential, we identified specific uses for wastewater surveillance in congregate settings. Although this framework helps capture and organize potential applications, most of them still reflect proposed rather than previously implemented use-cases, including: 1.1.1) monitoring the absence and early detection of outbreaks, 1.1.2) identification of hotspots during outbreaks, 1.1.3) enhancing outbreak control implementation, 1.1.4) evaluation of control effectiveness and outbreak resolution, and 1.1.5) optimizing design to reduce surveillance costs (Fig. 1). For each of these uses, the main benefits, challenges, modeling approaches, and key considerations are summarized in Table 1, and illustrative examples are presented in Table 2.

#### 1.1.1. Monitoring the absence of cases versus early detection of cases

The absence of a wastewater signal provides critical insight in congregate settings when monitoring infectious diseases that are not expected to be endemic but instead introduced from external sources. Even for endemic pathogens, such as SARS-CoV-2, disease transmission in congregate settings is characterized by periods of no detected cases, interrupted by intermittent detection. Thus, one of the primary applications of wastewater surveillance in these settings is determining the absence of a pathogen rather than solely monitoring increasing or decreasing concentration trends, as is commonly done in community-based wastewater surveillance programs.

In addition to the value of negative signals, wastewater can also support early case detection by identifying acute shifts in signal behavior that reflect new case introductions from new residents, staff, or visitors. Such changes may occur during the presymptomatic or early infectious stages, when shedding occurs before clinical symptoms appear. These detectable increases in signal can suggest the transition from absence of infection to early transmission, providing facilities with a window to intervene before symptomatic cases accumulate and become an outbreak, or to detect an asymptomatic burden that would otherwise go unnoticed without extensive testing.

Despite the reported variability, works assessing the lag time offered by wastewater surveillance indicate that signals precede clinical case reports by several days to weeks (Li et al., 2023; Shah et al., 2022). Evidence specific to congregate settings further suggests that wastewater monitoring can provide a similar lead time before confirmed cases are detected (Davó et al., 2021; Gamage et al., 2024; Keck et al., 2023; Piggott et al., 2023).

This operational value becomes especially important in settings with slow testing turnaround times, limited testing capacity, or high testing refusal rates (Beaudry et al., 2020). Examples include skilled nursing facilities where testing may be delayed due to patients' limited ability to report symptoms, and prisons where disincentives such as fear of stigma or isolation may discourage individuals from reporting symptoms (Beaudry et al., 2020; González-Montalvo et al., 2023). These early-warning roles can be operationalized using several modeling approaches designed to detect abrupt or gradual changes in wastewater signals (Table 1). These include:

##### 1.1.1.1. Change-point detection models.

These methods are used to identify points in time when the behavior of a system changes significantly, resulting in deviations from baseline viral signal levels that potentially indicate the introduction of new cases. Common techniques include Shewhart charts, which are effective for detecting abrupt changes using thresholds based on the mean and standard deviation, and Cumulative Sum or Exponentially Weighted Moving Average charts, which are designed to detect more gradual shifts by incorporating trends over time (Table 2) (Ocagli et al., 2025; Unkel et al., 2012).

##### 1.1.1.2. Regression models.

These models identify statistically significant deviations in wastewater signal over time. These tools can focus on estimating the expected signal intensity at each time point based on historical data and calculating standardized residuals (Unkel et al., 2012; Zareie et al., 2023). Large residuals suggest a meaningful departure from baseline conditions and may indicate the early stages of an outbreak.

#### 1.1.2. Identification of hotspots during an outbreak

When wastewater signals suggest an increase in the number of cases, the next step is to trace its origin and identify the specific location within the facility, which often requires improving spatial resolution through strategies such as upstream wastewater collection. This targeted approach can potentially enhance outbreak management, allows the identification of infection hotspots, and supports more efficient resource allocation by limiting unnecessary testing or non-targeted implementation of interventions.

The impact of wastewater surveillance on hotspot identification is especially relevant in some prisons, where thousands of residents may be housed in close proximity, and precise localization of cases is critical to maintaining normal operations in unaffected areas. Achieving finer spatial resolution has led several studies to leverage institutional layouts and dye-tracing techniques to guide upstream sampling from individual housing units and map internal wastewater flow patterns (Gamage et al., 2024; Karthikeyan et al., 2022; Kennedy et al., 2024).

Despite its potential, the implementation of upstream sampling faces several operational challenges. In many institutions, limited access to internal wastewater infrastructure prevents sampling at specific points, reducing the ability to obtain high spatial resolution. Additionally, the infrastructure within facilities complicates data interpretation by mixing multiple areas of interest before reaching the sampling point. Modeling approaches integrating wastewater data with location-specific screening, such as pooled testing by ward, floor, or housing unit, offer a promising alternative to improve case localization.

Spatial modeling can be used to detect non-random patterns in the geographic distribution. By evaluating spatial variation in disease occurrence, these models offer insight into contexts where transmission is influenced by place-based factors such as infrastructure, susceptibility, or characteristics of social organization (Chen et al., 2010). They are particularly valuable for targeting public health interventions where purely temporal analyses may be less sensitive to small but spatially concentrated signals. Similarly, spatiotemporal models can incorporate the temporal dimension to capture how clusters emerge, expand, or dissipate over time (Chen et al., 2010; Hu et al., 2025).

Applying classic spatial scan methods to wastewater surveillance requires special considerations compared to traditional epidemiological datasets (Chen et al., 2010; Unkel et al., 2012). Specifically, in wastewater-based applications, spatial units are not defined as arbitrary fractions of the population or predefined spatial windows but are determined by the sewer catchment structure. Each sub-catchment represents a discrete unit, and candidate clusters are formed from individual or aggregated catchments. These specifications can also be varied to assess robustness, for example, by testing alternative catchment aggregations or adjusting the expected signal based on resident population, population density, visitors, or wastewater flow. Examples of useful modeling approaches include:

##### 1.1.2.1. Spatial generalized linear mixed model.

These models estimate the detection probability or signal incidence across spatial units while adjusting for covariates and accounting for correlation between neighboring areas through shared random effects. They are particularly useful for quantifying spatial heterogeneity in risk and can incorporate contextual information to refine estimates. Residuals from the model help identify subunits where observed signals deviate from expectations, supporting early detection of localized anomalies (Chen et al., 2010).

##### 1.1.2.2. Spatial scan statistic.

The classical spatial scan statistic partitions the study area into candidate windows and compares the likelihood of the observed data under clustered and non-clustered assumptions, identifying areas where observed elevations are disproportionately higher than expected under a homogeneous distribution of cases (Table 2) (Chen et al., 2010; Kulldorff, 1997). In wastewater-based surveillance, this framework can be adapted by redefining spatial units according to individual or aggregated catchment areas and modeling the expected signal as a function of the resident population, wastewater flow, or shedding characteristics of subgroups.

##### 1.1.2.3. Bayesian spatio-temporal models.

These models estimate the probability that the wastewater signal in each catchment and time point exceeds a threshold, given observed concentrations. Observations are related to latent infection levels through a probability model (e.g., Poisson or log-normal) that incorporates covariates and measurement error as sources of variation, together with spatial and temporal structures. Priors capture parameter uncertainty, and Bayesian inference yields posterior probabilities of outbreak risk that help distinguish true transmission patterns across space and time (Kim et al., 2024).

##### 1.1.2.4. Network flow models.

These models treat sewer systems as dynamic networks in which wastewater carrying pathogen signals move through interconnected nodes and pipes before reaching sampling sites (Calle et al., 2021). This approach uses data from pathogen concentration, water use, and flow direction to model how flows from different buildings converge and dilute along the network. Thus, it enables the identification of where sampling will be most informative and how a positive signal at a specific site can be traced back to its upstream sources.

#### 1.1.3. Enhancing outbreak control

Implementing control measures is particularly challenging, as they must be deployed early and ideally only in scenarios with emerging outbreak activity. However, wastewater surveillance can enhance outbreak control by enabling the optimal deployment of interventions and providing a means to assess their impact.

One challenge is determining when a wastewater signal truly indicates outbreak risk, as it does not always correspond to active or persistent transmission. Control measures should only be implemented when changes in the signal suggest a high likelihood of an outbreak, while avoiding false positives driven by background noise or sporadic, self-limited infections. Conversely, false negatives may delay detection and lead to missed opportunities for control.

Regarding the types and early use of control measures, while proactive strategies such as health education and vaccination would be ideal for preventing outbreaks before they occur, in practice, these are not always feasible. In such situations, timely reactive measures, such as distributing protective equipment, implementing crowd control, increasing testing, and applying isolation and quarantine, play a crucial role in containing transmission during the early stages of an outbreak.

It is also crucial that administrators consider any potential negative impact on residents' quality of life, beyond only the effect on decreasing transmission as the final outcome. For example, in correctional settings, some residents perceive quarantine protocols as punitive “lockdowns,” which can lead to reduced social interaction, limited physical activity, and adverse mental health outcomes (González-Montalvo et al., 2023).

Using modeling approaches with wastewater as an indicator of disease burden can help optimize the identification of potential outbreaks, assess appropriate thresholds for triggering control measures, and select the most effective interventions. Useful models include:

##### 1.1.3.1. Hidden Markov Models (HMM).

This approach assumes that wastewater signals fluctuate between hidden non-outbreak and outbreak states (Table 2). The relationship between these hidden states and wastewater signal can be represented by state-specific probability distributions (e.g., Normal, log-normal, or inverse Gaussian), potentially adjusted for covariates such as flow or rainfall. Based on these distributions, HMM estimates the probability that each observation belongs to a specific state. These probabilities are then combined with transition probabilities specified in a transition matrix that determine the chance of remaining in or switching between states. This prevents isolated high values from being misclassified as outbreaks, while consistent peaks in the signal increase the probability of transitioning to the outbreak state (Chen et al., 2010; Unkel et al., 2012).

##### 1.1.3.2. Machine learning classification models.

This approach can identify patterns associated with disease burden by analyzing historical wastewater data in combination with epidemiological features (Hu et al., 2025). A practical example is the use of random forest, an algorithm that aggregates multiple decision trees to distinguish outbreaks from non-outbreak periods (Hu et al., 2025). This method can incorporate wastewater signals along with factors such as rainfall, temperature, sampling characteristics, and reported cases, assigning their relative importance as predictors and facilitating short-term forecasting of outbreak trajectories (Cho et al., 2023). Gradient boosted trees extend this idea by building trees sequentially, with each new tree correcting the errors of the previous ones (Cho et al., 2023). These are particularly effective at leveraging multiple correlated features to improve predictive accuracy under noisy surveillance conditions. Likewise, neural networks are models that learn flexible, weighted functions to combine predictors, rather than relying on rule-based splits as in tree-based methods. Particularly, recurrent neural networks (RNNs), such as long short-term memory (LSTM) models, extend this framework by incorporating information from previous days to detect patterns, including sustained increases or delayed peaks (Ai et al., 2022). A key limitation, however, is that in settings with low sampling frequency and limited contextual data, the application and performance of these big data approaches may be constrained.

##### 1.1.3.3. Compartmental models.

These models provide a structured framework for simulating disease spread and clinical trajectories, supporting the estimation of key parameters such as the reproduction number, enabling real-time identification of scenarios in which interventions should be deployed, and assessing their impact on epidemic dynamics and outbreak resolution (Huang and Morris, 2025; Blumberg et al., 2022). Wastewater data can be used to calibrate models by assigning shedding coefficients to infectious populations. The wastewater signal at time t can be expressed as the sum of infected individuals in each group multiplied by their average shedding contribution:$\text{W}(\text{t})=\sum_{i}\alpha_{i}\cdot N_{i}(t)$, where ${\text{N}}_{\text{i}}(\text{t})$ is the number of individuals in stage i (e.g., exposed, asymptomatic, symptomatic) and $\alpha_{\text{i}}$ is their corresponding shedding coefficient. Wastewater data can provide added value compared to clinical case data by capturing symptomatic and asymptomatic infections and potentially detecting changes earlier than case reports. Wastewater surveillance data can also be used in conjunction with case data to calibrate models.

#### 1.1.4. Evaluating the effectiveness of control measures and assessing outbreak course

After implementing control measures, wastewater can be used to assess their effectiveness and determine whether measures should be escalated, maintained, or relaxed based on the signal changes that suggest ongoing transmission risk. However, interpretation can be complicated by heterogeneous residual signals from infected or recovered individuals with active shedding and ancillary contributions, such as shedding from visitors or signal mixing from nearby residences (Gupta et al., 2020; Sims and Kasprzyk-Hordern, 2020). Wastewater use as a surrogate marker to quantify intervention effectiveness remains a relatively unexplored area in the literature, particularly for assessing the impact of this type of surveillance on public health metrics like cases averted through its implementation.

Initial evidence supports the feasibility of wastewater signal as a proxy to quantify the effect of control measures. For example, marked reductions in SARS-CoV-2 concentrations were observed in wastewater during COVID-19 vaccination campaigns in living facilities (Brenner et al., 2024). Although it is difficult to attribute this decline solely to immunization, these findings underscore the potential of wastewater surveillance for evaluating the impact of control interventions.

Models addressing these challenges should focus on identifying signal changes during an active outbreak to explore scenarios where adjustments in control measures are needed or where new interventions require evaluation to determine their potential impact. Useful approaches include:

##### 1.1.4.1. Time-series forecasting models.

Approaches such as ARIMA, SARIMA, and Prophet models can be applied during active outbreaks to assess whether signal intensity deviates from expected trends. These models can use pre-intervention historical patterns to generate short-term forecasts that serve as a counterfactual for the post-intervention period. This enables assessment of whether observed changes, such as abrupt increases or gradual declines, reflect the potential impact of control measures and inform operational planning for testing frequency, isolation capacity, and protective equipment allocation (Malki et al., 2021; Maria Jones and Winster, 2021).

##### 1.1.4.2. Regression models.

These models can help assess whether an intervention is associated with changes in the wastewater signal. For instance, interrupted time series regression can be specified as a segmented linear model, where the wastewater signal is represented as an intercept $\left({\text{β}}_{0}\right)$, a pre-intervention slope $\left({\text{β}}_{2}\right)$, an immediate level change at the time of intervention $\left({\text{β}}_{2}\right)$, a post-intervention change in slope $\left({\text{β}}_{3}\right)$, and an error term (ε) (Table 2). This framework offers the possibility of estimating the short-term effect $\left({\text{β}}_{2}\right)$ and long-term effect $\left({\text{β}}_{3}\right)$ of the intervention and generates a counterfactual series from the pre-intervention slope to compare with observed values. The effect size of the intervention can be estimated as a simple attributable effect, expressed as 1 – (observed/expected), thereby allowing assessment of whether changes in the wastewater signal are consistent with the intervention (Travis-Lumer et al., 2022). In addition, to account for residual shedding from previously infected individuals when assessing control measures, a decay component can be added to the model and parameterized as an exponential decay, a log-normal function, or another empirically informed distribution of shedding duration.

##### 1.1.4.3. Bayesian hierarchical models.

These models can estimate the posterior probability that the true wastewater signal has fallen below a predefined action threshold or can provide transmission-based estimates, such as the effective reproduction number, while accounting for measurement error and day-to-day variability. They can also incorporate intervention effects as a covariate that influences the underlying signal (Jin et al., 2024). Contributions from residual shedding can be incorporated by specifying latent components with priors on shedding duration (e.g., exponential, log-normal, or gamma). Modeling the decline in viral concentration as a probabilistic process rather than a fixed trend provides a statistical framework for determining whether control measures should be maintained, escalated, or relaxed.

##### 1.1.4.4. Agent-based models.

This approach simulates the heterogeneity of behavior, transmission, and clinical trajectories at an individual level (Huang and Morris, 2025). These models can be used to simulate changes in disease burden for different intervention strategies that can be implemented in response to wastewater signals (Hoover et al., 2023). In this framework, each simulated individual can contribute to the wastewater signal according to their infection or colonization status. The aggregated shedding across all agents can be compared with observed wastewater data to calibrate simulation parameters and evaluate the potential for earlier outbreak detection. Similarly, due to limited population movement in congregate facilities, increases in wastewater signals are shaped mainly by infections that arise and persist within the facility during the shedding period. This structure allows individual-level processes, such as institutional movements governed by controlled schedules or activities, to be represented more explicitly than in large, open populations.

#### 1.1.5. Optimizing surveillance design to reduce costs

Modeling has an indirect economic impact by facilitating the efficient implementation of wastewater surveillance, which translates into actionable epidemiological insights that can reduce costs by decreasing the magnitude, duration, and consequences of outbreaks. In addition to this effect on cost reduction, modeling also has a broader intrinsic role by optimizing surveillance system design through cost-effectiveness analysis approaches, such as sampling strategies, testing frequencies, and other design choices that improve overall resource utilization (Sanjak et al., 2024).

Compared to individual testing, wastewater surveillance could reduce costs by minimizing the number of individual tests, enabling targeted and timely interventions, and allowing detection of multiple pathogens in one sample (Pang et al., 2024; Rao et al., 2024; Yoo et al., 2023). For example, compared with clinical surveillance, cost modeling has found potential net benefits of around $172,000 at a single long-term care facility, and around $3.5–41 million at the national level in Japan during 4 weeks with a high incidence of COVID-19 (Yoo et al., 2025). This economic impact could be greater, as demonstrated by certain wastewater surveillance systems that enable field testing and avoid laboratory sample processing, which have shown a 6.5-fold cost reduction compared to clinical testing and approximately 50 % lower costs relative to standard community-based wastewater surveillance protocols (Cheng et al., 2025).

A further consideration is protocol optimization, which plays an important role in the implementation of wastewater surveillance. Previous work has shown that most associated costs stem from field sampling, including manhole access, sample collection, and viral load testing, suggesting that adjustments to these processes could substantially reduce expenses. In practice, reducing the number of sampling locations by 85 % has been shown to maintain a high degree of reliability while lowering costs by 80 % (Lo et al., 2025). Similarly, analytical and simulation models of two interacting patches have shown that, when interaction is high and setup costs are substantial, testing one site more frequently can be more cost-effective than testing both less often, assuming adequate surveillance sensitivity and specificity (Impalli et al., 2025). Modeling approaches useful for optimization should offer the ability to compare the diverse protocol configurations that may be implemented in these settings, including:

##### 1.1.5.1. Cost-effectiveness and cost-benefit analysis models.

These models evaluate the financial viability of wastewater surveillance compared to traditional surveillance by quantifying both direct and indirect costs, including the economic value of disease averted and resource optimization during implementation (Table 2). Direct costs include testing, equipment, and other operational resources needed to implement control interventions. Indirect costs include medical expenses for infected residents and associated sick leave among infected staff (Sanjak et al., 2024).

### 1.2. Expanding wastewater surveillance beyond SARS-CoV-2 in congregate settings

Despite these potential applications, most studies on wastewater surveillance in congregate settings focus on SARS-CoV-2, with few addressing other pathogens. Given the elevated transmission risk in these high-density environments, expanding surveillance targets to organisms such as *C. difficile*, *M. tuberculosis*, influenza virus, *S. aureus*, hepatitis viruses, and foodborne pathogens could strengthen outbreak detection and support broader disease monitoring (Kendig et al., 2024; Nicolle, 2014). However, each pathogen requires distinct evaluation, as differences in shedding routes, duration, environmental stability, and wastewater detectability can influence signal interpretability and necessitate pathogen-specific modeling considerations (Table 3).

## 2. Discussion

Wastewater surveillance for monitoring and controlling infectious disease outbreaks in congregate settings offers valuable public health benefits. However, its effectiveness depends on reliably translating stochastic wastewater signals into evidence-based guidance for policymakers. Mathematical modeling plays a key role in translating wastewater signals into meaningful insights, with the potential to help detect outbreaks earlier, enhance and monitor intervention strategies, and allocate resources more efficiently.

Modeling can help navigate challenges, including reducing the impact of signal stochasticity and environmental variability on inference. Beyond detection, predictive modeling can be applied to anticipate outbreaks, measure the impact of control efforts, and improve cost-effectiveness in disease management. Notably, while our approach aims to classify model applications into specific use cases, it is essential to recognize that these models are not strictly confined to a single use, with multilevel or hybrid approaches potentially enhancing these applications by improving estimates of disease burden.

Successful implementation of wastewater-based surveillance involves the incorporation of robust data-driven modeling approaches but also relies on strong institutional support and well-defined policy frameworks to facilitate coordination between public health authorities, facility administrators, and staff. Shifting resources from individual testing to wastewater surveillance is not always straightforward, even when wastewater offers clear advantages for decision-making. In practice, the absence of clear policies and coordinated collaboration often leads to uneven implementation.

## 3. Conclusion

Wastewater surveillance complements traditional outbreak detection strategies and provides a powerful tool for real-time decision-making in settings where clinical surveillance is limited. Although the potential to reduce the burden of outbreaks in congregate settings is of clear relevance to residents of these facilities, this also has implications for spillover transmission to the general community. Expanding wastewater surveillance to a broader range of pathogens and enhancing analytical methods could improve disease tracking, enhance prevention, and reduce the disease burden in these settings.

## Figures and Tables

**Table 1:** Benefits, challenges, and modeling opportunities for wastewater surveillance of infectious diseases in congregate settings.

Applications and benefits	Challenges	Modeling approaches	Key considerations for modeling approaches
**Monitoring the absence of cases versus early detection of cases.** • Allows confirmation of pathogen absence, reducing the use of routine surveillance or interventions. • Facilitates early detection of signal increases.	• Poor signal detection at low infection levels. • Difficulty distinguishing asymptomatic shedding from early transmission.	**Change-Point Detection Models (Control charts):** Detect sudden shifts in wastewater signals indicating pathogen introduction. **Regression Models:** Estimate expected disease trends using historical data and detect deviations to set alert thresholds.	**Control charts:** Most useful when frequent wastewater signal testing is available **Regression models:** Useful when sufficient historical data exist to define trends. Flexible enough to incorporate external and internal sources of signal variability.
**Identification of hotspots during an outbreak:** • Clarifies the need for population-wide versus localized control. • Reduces unnecessary testing and optimizes resource allocation. • Supports targeted control measures.	• Wastewater sampling points may not perfectly correspond to the target population, and cross-contamination from shared sewer systems can affect results. • There may be limited options for upstream wastewater sampling, limiting the opportunity for fine-scale measurements.	**Spatial Generalized Linear Mixed Models:** Estimate detection probability or rate across spatial units, adjusting for covariates and spatial correlation using random effects. **Spatial Scan Statistic:** Evaluates windows defined by sewer catchments to identify where signal is significantly higher than expected under a homogeneous distribution. **Bayesian Spatiotemporal Models:** Estimate infection probability over space and time, accounting for uncertainty and data variability. **Network Flow Models:** Simulate wastewater pathways to trace outbreak origins and predict spread.	**Spatial Generalized Linear Mixed Models**: Best suited when there is adequate spatial coverage and covariate data **Spatial Scan Statistic:** Good when catchment population, wastewater flow, or data are available to estimate each catchment’s expected contribution. **Bayesian Spatiotemporal Models**: More appropriate if uncertainty is high (limited sampling, heterogeneous shedding). **Network Flow Models:** Most useful when the sewer network is well characterized, as they rely on detailed maps and assumptions about flow.
**Enhancing outbreak control:** • Allows early implementation due to lead time of signal detection. • Identifies scenarios when control measures should be deployed. • Decreases the size/severity of outbreaks.	• Wastewater detection does not always indicate active transmission or a potential outbreak.	**Hidden Markov Models (HMM):** Probabilistic approach to detect outbreaks by estimating transitions between latent states (outbreak vs non-outbreak), based on observed wastewater signals and emission distributions. **Machine Learning Classification Models:** Differentiate outbreaks from spurious wastewater fluctuations, supporting data-driven decisions on activating control measures. **Compartmental Models:** Simulate disease spread and different scenarios for disease control.	**HMM:** More appropriate when signals are noisy or irregular. They smooth variability due to incorporation of latent states. **Machine learning classifiers:** Best when analysis involves large training datasets. **Compartmental models:** Useful for exploring general outbreak trajectories and broad interventions.
**Evaluating the effectiveness of control measures and assessing outbreak course** • Informs decisions on escalating or relaxing control measures • Allows for comparison of different control strategies and selection of the most appropriate control intervention	• Difficulty in distinguishing continued viral shedding from recovered individuals and active transmission. • Contributions to wastewater signal from visitors, staff, and contamination from nearby properties can obscure outbreak resolution. • Quantifying trends in wastewater signal is limited by biological (shedding patterns) and logistical (sampling frequency) variability.	**Time-Series Forecasting Models:** Predict outbreak progression using wastewater trends during implementation of control measures. **Regression Models:** Assess whether an intervention is associated with changes in wastewater viral load by comparing post-intervention observations to the expected trend based on pre-intervention data. **Bayesian Hierarchical Models:** Estimate the probability that the actual viral load is below a threshold indicating outbreak resolution, accounting for variability to inform control measures. **Agent-Based Models:** Simulate intervention effects, considering individual behavioral and biological heterogeneity.	**Forecasting Models**: Depend on sufficient availability of pre-intervention data. **Regression Models (Interrupted time series):** Best performance when the pre-intervention baseline is stable and interventions are discrete. **Bayesian Hierarchical Models:** Useful when data are noisy, sparse, or when there is interest in quantifying uncertainty around outbreak resolution. **Agent-Based Models:** Most appropriate when detailed data on individual behavior and interactions are available.
**Optimizing design to reduce surveillance costs:** • Cost-optimization and cost-effectiveness assessment of wastewater surveillance.	• Balancing cost savings in scenarios with uncertain surveillance accuracy. • Dependence of cost-effectiveness on incidence, facility size, and sampling design. • Uncertain effectiveness of outbreak control on outbreak size reduction.	**Optimization Models:** Identify the most efficient sampling frequency and coverage to maximize epidemiological value per cost. **Cost-Effectiveness Models:** Assess financial trade-offs between wastewater surveillance and alternatives, such as relying on syndromic surveillance.	**Optimization and cost**-**effectiveness models:** Well suited when surveillance accuracy and outcome data are available to compare wastewater surveillance with alternative surveillance systems and methodologies (e.g., sampling strategies).

**Table 2:** Illustrative modeling frameworks supporting applications of wastewater surveillance.

Practical use	Modeling approach	Explanation
**Monitoring the absence of cases vs. early detection of cases**	**CUSUM control chart** ${\text{C}}_{\text{t}}=\mathrm{Max}\left(0,\;C_{t}-1\;+\frac{\left(Y_{t}-\mu \right)}{\sigma }-\text{k}\right)$ ${\text{Y}}_{\text{t}}$: observed wastewater measurement on day t μ: expected baseline mean in the absence of outbreaks σ: baseline standard deviation k: minimum shift (in SD units) that the chart is designed to detect. An alarm is raised when C_t_ exceeds a decision threshold.	When Y_t_ is close to the baseline mean, the standardized deviation is near zero and the statistic stays small. Higher values of Y_t_ cause the decision threshold to be reached, at which point an alarm is triggered (C_t_≥h, where h is the decision threshold). The max (0) operator resets the chart to zero when values fall below baseline, ensuring that only sustained increases, not isolated spikes, generate sustained alerts.
**Identification of hotspots during an outbreak**	**Scan Statistic (population-weighted)** $\frac{L(Z)}{L_{0}}={\left(\frac{n_{z}}{\mu (Z)}\right)}^{n_{z}}{\left(\frac{N-n_{z}}{N-\mu (Z)}\right)}^{N-n_{z}},\text{μ}(\text{Z})=\text{N}\left(\frac{Population\;in\;Z}{\text{Total population}}\right)$ **L(Z):** likelihood under the alternative hypothesis (Z contains a cluster). **L**_**0**_: likelihood under the null hypothesis (no cluster, homogeneous distribution). **n**_**z**_: wastewater signal observed in the sub-area Z **μ(Z):** expected signal in Z under homogeneous distribution, estimated as the total wastewater signal N multiplied by the proportion of the population in Z represented by the catchment site. **N:** total wastewater signal observed across the entire system during the study period.	This method evaluates whether the wastewater signal in a given ward or building is disproportionately elevated compared to what would be expected if cases were distributed proportionally to the population. The numerator L(Z) reflects the likelihood of the observed data under the assumption of clustered signal, while the denominator L_**0**_ reflects the likelihood under the homogeneous expectation. Their ratio indicates how much more strongly the “cluster” explanation fits the data. The formula is derived assuming a Poisson model. Wastewater signals can be normalized to a dimensionless scale by quantifying them relative to the expected contribution of a single shedding individual.
**Enhancing outbreak control**	**Two-state Hidden Markov Model (HMM)** $\text{P}\left({\text{O}}_{\text{t}}|g_{\text{t}}\right)=\frac{\pi_{t-1}f_{O}\left(g_{t}\right)}{\pi_{t-1}f_{O}\left(g_{t}\right)+\left(1-\pi_{t-1}\right)f_{N}\left(g_{t}\right)}$ $\pi_{t-1}$: outbreak probability on the previous day. This incorporates transition probabilities between outbreak and non-outbreak states. $g_{t}:{\text{W}}_{t}-{\text{W}}_{\text{t}-1}$: daily change in the wastewater signal $f_{o}\left(g_{t}\right)$: likelihood of the observed change if in the outbreak state (distribution centered on positive growth). $f_{N}\left(g_{t}\right)$: likelihood of the observed change if in the non-outbreak state (distribution centered near zero). Decision rule: trigger an alarm if $\text{P}\left({\text{O}}_{\text{t}}|{\text{g}}_{\text{t}}\right)$ exceeds a threshold for r consecutive days.	This model assumes that the wastewater signal can arise from two hidden states: non-outbreak (small day-to-day fluctuations) and outbreak (sustained increases). Each state is given a probability distribution that describes how daily changes in wastewater usually look under that condition. As new data are observed, Bayes’ rule is used to update the probability of being in each state. In practice, this means the model continuously estimates whether today’s signal is more or less consistent with an outbreak.
**Evaluating the effectiveness of control measures and assessing outbreak course**	**Interrupted Time Series (ITS) model** $\log\left({\text{W}}_{\text{t}}\right)={\text{β}}_{\text{0}}\text{+}{\text{β}}_{\text{1}}\text{t+}{\text{β}}_{\text{2}}{\text{I}}_{\text{t}}\text{+}{\text{β}}_{\text{3}}\left(\text{t}-{\text{T}}^{*}\right){\text{I}}_{\text{t}}+{\text{ε}}_{\text{t}}$ W_t_: wastewater measurement at time t (days, weeks) I_t_: indicator (0 before intervention, 1 at and after intervention at time T*). β_0_: baseline level at time zero. β_1_: baseline slope before intervention. β_2_: immediate level change after intervention. β_3_: change in slope after intervention. ε_t_: error term.	This model separates the wastewater time series into two segments, before and after the intervention, and estimates whether there is an immediate change in level (β_2_) and/or a change in slope (β_3_). A negative value of β_2_ indicates an immediate reduction in the wastewater signal at the moment of intervention, while a negative β_3_ indicates that the long-term trend decreased relative to the pre-intervention period.
**Reduction of surveillance costs**	**Cost optimization model** $\text{C}(\text{f})={\text{C}}_{\text{s}}\cdot \text{f}+\sum_{t}{\text{C}}_{0}(\text{t})\cdot \text{P}(\text{t}|\text{f})$ C(f) = total expected cost under sampling frequency f. f: testing frequency (e.g., samples per week). C_s_: cost per wastewater sample. C_o_(t): cost associated with detecting the outbreak with a delay of t days. P (t|f): probability that the detection delay is t days given a sampling frequency f.	This framework balances the cost of wastewater sampling against the cost of delayed outbreak detection. Increasing the sampling frequency (f) raises expenses but shortens the average time to detection, while less frequent sampling reduces testing costs but prolongs outbreak lead times. The optimal frequency is the one that minimizes the total expected cost. The detection delay can be estimated from empirical data, simulations, or approximated based on the sampling schedule.

**Table 3:** Characteristics of pathogens potentially relevant for wastewater monitoring in congregate environments.

Pathogen	Key biological features
**SARS-CoV-2** (Kallem et al., 2023) (Patel et al., 2021) (Jones et al., 2020)	**Main shedding route and duration:** Respiratory and fecal (reported in 30–60 % of cases); detectable up to several weeks and as early as 3–5 days before the onset of classic symptoms, with variation by disease severity and viral variant. **Detectability in wastewater:** Highly variable across contexts but generally high (12–100 %). **Environmental persistence:** Moderate; depending on the surface, the virus can remain detectable from minutes to several weeks. In sewage, it can remain detectable for several days to weeks **Potential use**: Early outbreak detection and short-term forecasting in congregate facilities.
***Clostridioides difficile*** (Kramer et al., 2006) (Xu et al., 2014)	**Main shedding route and duration:** Fecal (spores); shedding can be prolonged in asymptomatic carriers. **Detectability in wastewater:** Studies reported high detection frequency in raw sewage influent (up to 90–92 % in some studies) **Environmental persistence:** Very high, with spores remaining viable for months. **Potential use**: Monitoring prevalence of colonization and infection at the facility level.
***Mycobacterium tuberculosis*** (Mtetwa et al., 2022a) (Mtetwa et al., 2022b) (Kramer et al., 2006)	**Main shedding route and duration:** Respiratory; shedding can persist for months in active cases. Fecal shedding has been observed in extrapulmonary infections. Approximately 20 % of patients with pulmonary disease may also have extrapulmonary involvement. **Presence in wastewater:** Detection varies by species but consistently high, approaching 100 % for *M. tuberculosis* complex and 75–100 % for *M. tuberculosis* in some studies. **Environmental persistence:** Ranging from days to months; high persistence primarily in soil, urine, feces, and water. **Potential use:** Detecting presence as a sentinel for ongoing or hidden transmission.
**Methicillin-resistant *Staphylococcus aureus*** (Al-Mustapha et al., 2024) (Goldstein et al., 2012) (Kramer et al., 2006)	**Main shedding route and duration:** Nasal and dermal, fecal shedding has been reported but is less common; shedding is variable and intermittent but can persist for months in persistent carriers if not eradicated. **Detectability in wastewater:** Less validated but reported between 28 % and 50 %. **Environmental persistence:** Typically, days to weeks. **Potential use**: Identifying reservoirs of resistant strains and sustained carriage within institutions.
**Influenza virus** (Toribio-Avedillo et al., 2023) (Lowry et al., 2023) (Zafeiriadou et al., 2024) (Kramer et al., 2006)	**Main shedding route and duration:** Respiratory (sputum, secretions); shedding can persist for days but can be prolonged in certain cases. Fecal shedding has been reported in some strains (~36 %) but is not a primary route. **Frequency of detectability in wastewater:** Varies across reports. Overall prevalence ranges from 11–43 % for influenza A and 1–51 % for influenza B. **Environmental persistence:** Persistence depends on pathogenicity, typically lasting for several days. **Potential use**: Detection of seasonal outbreaks and monitoring of epidemic peaks in closed settings.
**Hepatitis A/E virus** (Zulli et al., 2024) (Raya et al., 2024) (Kramer et al., 2006)	**Main shedding route and duration:** Fecal shedding; can persist for weeks to months. **Detectability in wastewater:** Hepatitis A virus has been reported in 14–51 % of samples, while hepatitis E virus has been reported in 2–24 % of samples in studies from Asia and America. **Environmental persistence:** Days to weeks. **Potential use**: Early detection of introductions to prevent large outbreaks in closed communities.
**Norovirus** (Huang et al., 2022)(Kramer et al., 2006)(Guzman et al., 2025)	**Main shedding route and duration:** Fecal; shedding can be prolonged in some individuals and may persist for weeks after symptoms have subsided. **Detectability in wastewater:** High, with RNA frequently detected (~82 %). **Environmental persistence:** Hours to days on surfaces. It can persist for days in wastewater, with an estimated decay rate of 0.02–0.21 *d*^− *1*^ **Potential use**: Detection of sudden gastroenteritis outbreaks and persistence in facilities.

Detectability in wastewater refers to the detection results reported in the cited manuscripts, rather than the probability of detection given pathogen presence. Environmental persistence includes surface data due to limited wastewater-specific estimates.

## Data Availability

No data was used for the research described in the article.
